# Intraocular Microsurgical Forceps (20, 23, and 25 gauge) Membrane Peeling Forces Assessment

**DOI:** 10.1155/2013/784172

**Published:** 2013-06-24

**Authors:** Raul Velez-Montoya, Chirag Patel, Scott C. N. Oliver, Hugo Quiroz-Mercado, Naresh Mandava, Jeffrey L. Olson

**Affiliations:** ^1^Department of Ophthalmology, University of Colorado School of Medicine, Rocky Mountain Lions Eye Institute, Aurora, CO 80045, USA; ^2^Department of Ophthalmology, Denver Health Medical Center, University of Colorado School of Medicine, Denver, CO 80204, USA

## Abstract

*Background*. To assess the peeling forces exerted by different calibers of microsurgical forceps on an experimental model of epiretinal membrane. *Methods*. A model of epiretinal membrane was constructed using thin cellulose paper and heptanes-isopropyl alcohol 1% mixture. The model was mounted on a force censoring device. Subsequently, flaps were created with three different microsurgical forceps of different calibers. We recorded the number of attempts, the duration of the event, and the pushing and the pulling forces during the peeling. The results were compared by a one-way ANOVA and a Fisher unprotected least significant difference test with an alpha value of 0.05 for statistically significance. *Results*. There was a statistical significant difference on the pulling and pushing forces between the 25 gauge (13.79 mN; −13.27 mN) and the 23 (6.63 mN; −5.76 mN) and 20 (5.02 mN; −5.30 mN) gauge, being greater in the first (*P* < 0.001). There were no differences in the duration of all events, meaning that all the forces were measured within the same period of time. *Conclusions*. The 25 gauge microsurgical forceps exerted the greatest mechanical stress over our simulated epiretinal membrane model and required more attempts to create a surgical suitable flap. The clinical implication of this finding is still to be determined.

## 1. Introduction

The surgical resolution of vitreoretinal diseases involves the micromanipulation of very fragile structures. A successful surgery depends upon the surgeon possessing a particular set of skills that include precise manual dexterity, fine visual-motor coordination, and improvisation capabilities, acquired after long hours of training [1, 2]. Imprecise movements due to tremor, poor visibility, and fatigue often may result in tissue damage which can be irreversible and sight-threatening depending on location [3, 4]. 

Macular surgery is one such scenario in which external factors (patient movements and surgical instruments), along with the surgeon's dexterity, may influence the outcome [5]. Macular hole (MH) repair, epiretinal membrane (ERM), and internal limiting membrane (ILM) peeling are perfect examples where the application of unknown forces to the tissues may lead to hemorrhage, tearing, and potential irreversible visual loss [5–7].

With the introduction of minimally invasive surgical techniques (23 and 25 gauge vitrectomy), macular diseases are addressed surgically more often and earlier than ever [5]. Along with the change in surgical paradigm, several aspects of retinal instruments have undergone further refinement [8, 9]. Microsurgical forceps (MSF) play a central role on macular surgery, since they allow fine surgical delamination, grasping, and manipulation of delicate structures such as ERMs and ILM. 

Since the ability to achieve surgical objectives during macular surgery is determined in part by the limits of the instruments used, we assessed the magnitude of peeling force that different sizes of MSF are able to exert over tissue during ocular surgery. Being aware of these forces and limit their impact on the retina, has the potential to improve precision and diminish surgical complications. To achieve this objective, we designed an experimental model simulating the peeling of an ERM, comparing the peeling forces from the three commercially available surgical calibers.

## 2. Methods

A model of macular surgery, simulating an ERM peeling, was constructed. In order to simulate the tissues characteristics, we selected materials that closely resemble the mobility and fragility of the structures, while keeping the same close relationship between them. For simulating retina we used a piece of 2 × 2 cm of extra-thin cellulose paper (cellophane paper, *≈*50 *μ*m ± 1.3 *μ*m). The cellophane sheet was cut and mounted over a pressure transducer (Pulse Transducer, ADInstruments, Colorado Springs, CO) which was connected to a data acquisition system (PowerLab 8/30 System, ADInstruments, Colorado Springs, CO). The cellophane sheet was a suitable simulated retina, being both mobile yet fragile when exposed to tractional forces. The pressure transducer was able to measure force in vectors: downward (pushing) and outward (pulling) exerted on the simulated retina. After calibration of the transducer by applying forces of known magnitude in the direction of both vectors, the results of the tests were directly measured from the force transducer. 

**Figure 1:**
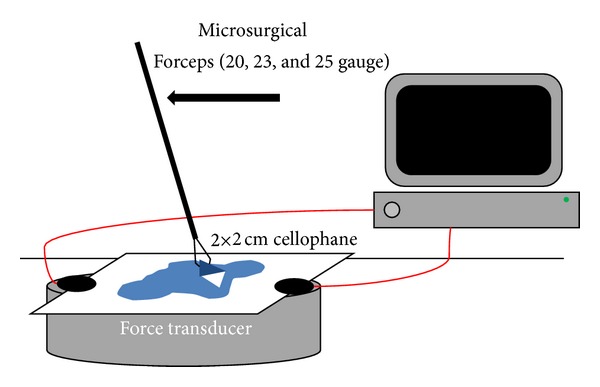
Graphic representation of the basic settings used for the experiment. The MSF used were brand new and commercially available.

To simulate an ERM and its close relationship with the retina, we dripped three drops of heptanes-isopropyl alcohol 1% mixture (rubber cement) over the cellophane paper surface and let it dry for 10 minutes prior to further manipulations (Figure 1). Similar to an ERM, an edge could then be identified and peeled.

**Figure 2:**
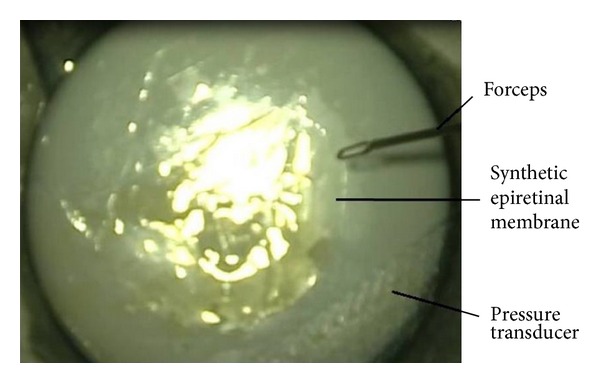
Microphotograph taken with the surgical microscope during one of the attempts to create a flap.

Brand new disposable 20, 23, and 25 gauge MSF were used for the simulated membrane peeling (Alcon Grieshaber-Switzerland/Alcon Labs, Inc., Fort Worth, TX). A flap on the simulated ERM was created a minimum of five times to ten times per model by the surgeon with all three MSF (CP). The experiment was conducted on three times per forceps caliber. For every attempt we recorded the number of attempts needed in order to create a suitable flap, the mean pushing and the mean pulling forces, exerted on the cellophane paper, and the duration of every attempt, defined as the time that the instrument was in contact with the artificial retina (Figure 2). 

Data is presented as median and standard error of measurement (SEM). Statistical analyses were made using and excel spreadsheet (Excel 2007; Microsoft Corp., Redmond, WA). A one-way ANOVA test was used to identify differences in the variability of the means among groups, using a *P* value of less than 0.05 for statistical significance. A Fisher unprotected least significant difference (FLSD) test was used to assess statistical difference between means within study groups.

## 3. Results

We constructed three models of experimental ERM peeling per caliber (9 models). In every model a minimum of five flaps were attempted: mean 7.5 ± 2 attempts (range 15 to 24). The mean ± SEM values of the pulling, pushing forces, duration and number of attempts to create a flap are summarized on Table 1. 

**Figure 3:**
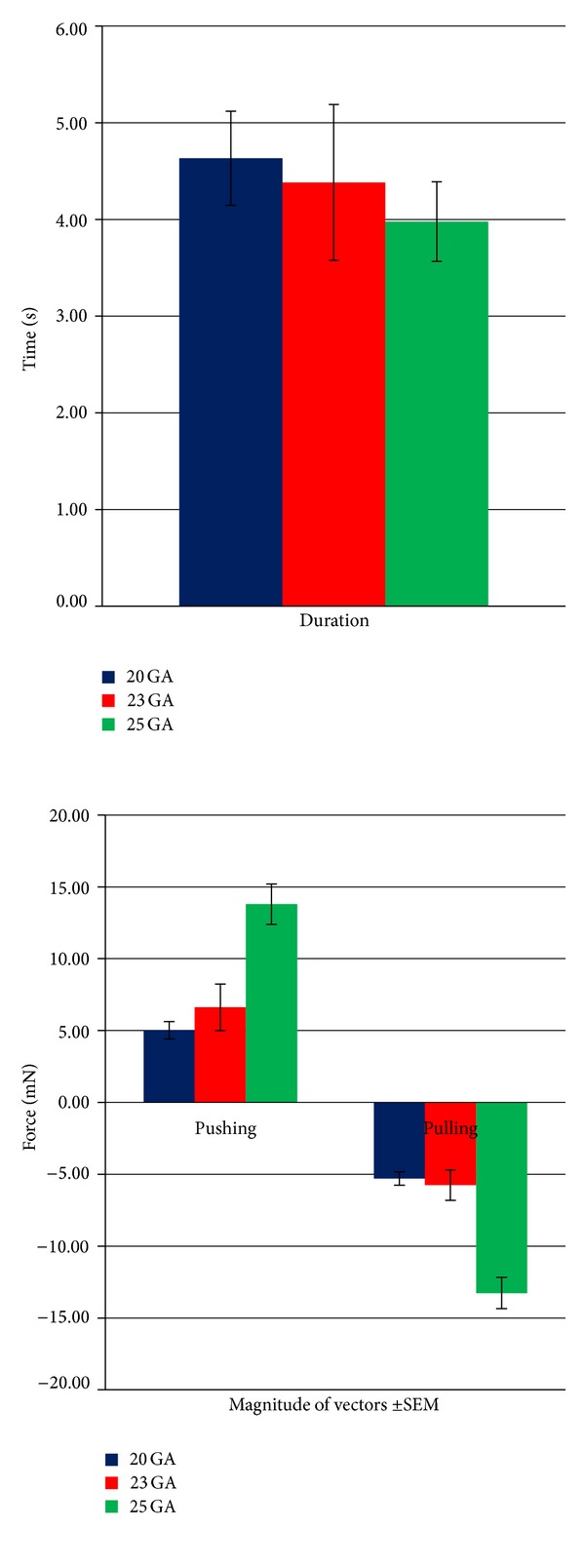
Graphic representation of the mean duration and principal vector forces with their corresponding standard error of measurement (SEM).

The duration of each attempt to create a flap was similar with all three calibers, with an overall mean duration of 4.33 ms (range 2.05 to 6.25 ms). The variance analysis showed that there was no significant difference in the duration of the attempts to create a flap among the three calibers, meaning that all other measured forces were applied over a similar period of time (*P* = 0.6) (Figure 3).

The 25 gauge MSF measured the strongest pushing and pulling forces: 13.79 mN and −13.27 mN, respectively, while the 20 and 23 gauge MSF had very similar measurements (5.02 mN; −5.30 mN and 6.63 mN; −5.76 mN, resp.). 

The ANOVA analysis showed that there was a statistical significant difference among the means of the pushing forces (*P* < 0.01). Further analysis with FLSD demonstrated that there was a significant difference between the mean pushing forces of the 20 gauge and 25 gauge MSF (*P* < 0.01) and between the 23 gauge and 25 gauge (*P* < 0.01), being in both cases the force's magnitude greater with the 25 gauge MSF. There was no statistical difference between the 20 and 23 gauge mean pushing forces (Figure 3). 

On the pulling analysis, the forces exerted with the 25 gauge MSF were again greater than with the 23 gauge (*P* < 0.01) or 20 gauge MSF (*P* < 0.01). There were no statistically significant differences on the pushing or pulling forces between the 20 and 23 gauge MSF (Figure 3). 

The number of attempts required to create a suitable flap on the simulated ERM was significantly greater with the 25 gauge MSF than with the other two calibers. 

## 4. Discussion

Macular procedures like MH repair and ERM peeling have become standard procedures among retinal specialist, mainly because current surgical techniques and available technologies allow good rates of success [5, 10]. Comparative studies between the standard 20 gauge vitrectomy and minimally invasive vitrectomy techniques have shown that there is no significant difference in terms of intraoperative and postoperative complications, which along with shorter intraoperative time, reduced postoperative discomfort and intraocular inflammation, have contributed in the rapid adoption of the new surgical paradigm [5, 7, 11, 12]. Proof of this change is that since 2007, the results of the Preferences and Trends survey (conducted by the American Society of Retinal Specialists among its members) show that 80% of the respondents use minimal invasive techniques for most of their cases (Mittra RS, Pollack JS. Preferences and Trends Survey. Poster presented at 25th Annual American Society of Retina Specialists Meeting, December 1–5, 2007; Indian Wells, CA). 

In this study we specifically investigated the amount of mechanical forces that MSF exert on retinal tissue. The results of our experimental model showed that mechanical forces of pushing and pulling are higher with smaller surgical instruments than with standard 20 gauge instruments, being significant only between the 20 gauge and 25 gauge (*P* < 0.01). Since the tip of the three MSF calibers was identical (corroborated with the manufacturer and after examination under microscope, data not shown), a possible explanation for this results is the difference on surgical shaft stiffness. It is important to note that although the rubber cement film kept a very close relationship with the cellulose paper (similar to a true ERM), it was not as thin as a real ERM. Therefore the forces needed to create a flap may be amplified. Another issue to consider is that given the extremely small forces involved, these differences may not be clinically relevant. For instance, the retinal adhesion force to the retinal pigment epithelium has been calculated to be around 140 mN, several times greater than the forces exerted by the MSF on this experiment [13, 14]. Therefore it is important to keep the correct perspective.

In a previous study, Gupta et al. recorded the axial tool shaft forces during retinal manipulation in vitro in a porcine cadaver eye model [1, 15]. He concluded that up to 75% of all measured forces were below 7.5 mN in magnitude [1, 16, 17]. Our results are consistent with those reported by Gupta (5.3 nM for a 20 gauge MSF). Despite the similar results it is important to highlight that in our study we measured the pulling and the pushing forces exerted directly by the tip of the instrument and that our experiment also included different instrument calibers that were not available at that time (23 and 25 gauge). On subsequent test of perception of forces, Gupta et al. found that only 19% of event at this force level were felt by the surgeon [1, 9, 16]. Our study results show that smaller MSF may be more traumatic to retinal tissue and the inability of the surgeon to feel most of these events may increase unintentional tissue damage theoretically. 

In summary, 25 gauge MSF registered greater pulling and pushing forces over a simulated model of retina ERM. The same caliber required more attempt to create a surgical suitable flap. There were no differences between the forces exerted neither by the 20 and 23 gauge MSF nor on the numbers of attempts to create a flap. Given the extremely low forces involved, it is still not clear if the differences are clinically significant. 

## Figures and Tables

**Table 1:** Mean forces and number of attempts.

Caliber	Duration (seconds)	Pushing (mN)	Pulling (mN)	Number of attemps
20 gauge	4.63 ± 1.62	5.02 ± 2.01	−5.30 ± 1.51	3.7 ± 1.01
23 gauge	4.38 ± 1.97	6.63 ± 3.96	−5.76 ± 2.58	2.0 ± 0.7
25 gauge	3.98 ± 1.93	13.79 ± 6.58	−13.27 ± 5.10	7.3 ± 1.58

Summary of the duration and mean ± standard deviation of the forces applied during the experiment. mN: millinewtons.
